# Children, HIV, emergencies and Sustainable Development Goals: roadblocks ahead and possible solutions

**DOI:** 10.1002/jia2.25046

**Published:** 2018-02-27

**Authors:** Dick Chamla, Chewe Luo, Priscilla Idele

**Affiliations:** ^1^ UNICEF Emergency Response Team, Health section New York NY USA; ^2^ UNICEF HIV Section New York NY USA; ^3^ UNICEF Data and Analytics section New York NY USA

**Keywords:** children, adolescents, HIV, SDG, climate change, emergencies

## 1 Introduction

**Figure 1:**
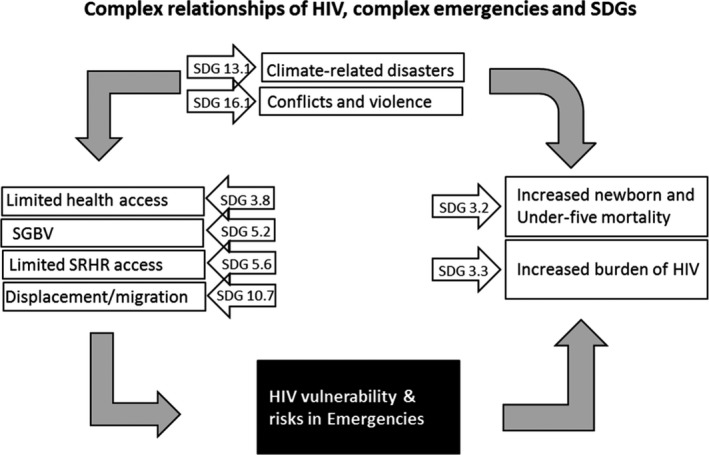
Complex relationship of HIV, emergencies and SDGs. SGBV, sexual and gender‐based violence; SRHR, sexual reproductive health and rights; SDG, Sustainable Development Goals.

Climate change, violent conflicts, and HIV/AIDS are linked to multiple Sustainable Development Goals (SDGs) through complex pathways (Figure 1) that include food insecurity, population displacements and migration, disruptions of health and HIV services, and increased incidences of sexual based violence. This interlinkage has the potential to result in high newborn and under five mortality rates and increased burden of HIV, directly affecting SDG 3.2 and 3.3 with children and adolescents being primarily affected.

In the past two years, five severe (classified by the UN as L3) emergencies were declared with over 50 million children caught up in major conflicts and other humanitarian crises 1. Nine of 21 countries deemed “high priority” for HIV by UNAIDS are fragile, conflict‐affected, or affected by climate‐related hazards. Today, more than 59 million people are displaced – 22 million more than a decade ago 2, while more than 70 million people in 45 countries are food insecure – 40% more than in 2015. There are more than 1.8 million people living with HIV in emergency settings, with children under the age of 15 years accounting for around 10% 3. Emergencies have increasingly become protracted (long term) with an average stay in refugee camps reaching 20 years 4 – implying that children could face more HIV risks throughout their adolescence in a refugee camp. The road towards the SDGs is further constrained by rapid population growth with an estimated one billion children likely to live in Africa by mid‐century, of which 217 million will be under‐five and over one third living in conflict‐affected zones 5.

Yet, most humanitarian plans and appeals have not been included in national development strategies, HIV interventions are largely underfunded despite high HIV vulnerability in emergency contexts 6, and most funding opportunities have been short term and focussed on immediate life‐saving interventions. Similarly, the opportunities provided by investments in SDGs are not optimal in humanitarian settings. These include medium‐long term funding by global financial instruments such as the Global Fund to Fight AIDS, Tuberculosis and Malaria (GFATM) and novel service delivery models, including enabled community systems that have had remarkable impact on improving HIV service delivery and access in countries such as South Africa 7.

These roadblocks, and a divide between humanitarian and development fields have tremendous implications for children and adolescents living with HIV in emergency contexts such that a clear way forward, building on the current global discourse on humanitarian‐development nexus, remains critical.

## 2 Implications for HIV

Most L3 emergencies have a potential to reverse gains in the global HIV response – including the legacy of the global plan on the elimination of new paediatric HIV infections. This five‐year plan was developed in 2011 with the aim of reducing new paediatric HIV infections by 90% and AIDS‐related maternal and paediatric mortality by 50% 11. Over the course of the Global Plan, more than 60% of annual new HIV infections were reduced, translating to 1.2 million new infections among children averted 8. However, among countries affected by emergencies such as Nigeria, the reduction in new infections remains as low as 40% 8. The effects of climate change are more profound in Africa and Asia, where there is a disproportionately high burden of HIV. Of the nine fragile countries with high HIV burden, five are in Central and West Africa, contributing 45% of the global number of new paediatric HIV infections with Nigeria alone accounting for more than 27% 9.

As more fragile countries have graduated to middle income status, development assistance for HIV will likely reduce, as for instance, domestic contribution thresholds by eligible middle income countries for grants from the GFATM is 20% to 60% – a significant rise from 5% for developing economies 10. Without significant financial resources, these fragile states will not be able to sustain their HIV response.

## 3 Way forward: humanitarian‐development nexus and HIV response

The humanitarian‐development nexus provides an important framework that could bridge a divide between these two fields that are guided by two separate, but complementary global processes – Agenda 2030 for sustainable development 16 and Agenda for Humanity 17, endorsed during the World Humanitarian Summit in 2016. The nexus calls for joint analysis and planning; defining collective outcomes; and joined‐up programming 18. Adapting this nexus in HIV responses during the emergencies could provide a way forward for addressing the roadblocks previously mentioned. The first step could be understanding the climate change or conflict risks that could impact HIV response through a joint analysis using current evidence and existing analytical frameworks. It is also important to note during the analysis that different types of emergencies such as *acute* or *protracted*, could have different needs requiring different programme designs. This risk analysis could form the basis of risk‐informed joint planning, allowing synergies between HIV and humanitarian interventions.

Joined‐up programming could be facilitated with sustainable financing by optimizing development and humanitarian funding mechanisms 14, and ensuring systematic integration and transition of humanitarian interventions to national or local authorities. Linking early recovery, resilience building and health system strengthening will ensure a quick development pathway after an emergency. Empowering communities and their infrastructure is equally important for resilience and improved outcomes. There is mounting evidence of the importance of community health workers in the reduction in under‐5 mortality rate 15 that could also benefit children and adolescents living with HIV. Some promising practices are also emerging such as GFATM's establishment of a special envelope for “challenging operating environments” 19 that aims to expand access to services in manmade or natural crises. New models of service delivery and innovations such as Point‐of‐Care diagnostics for dual HIV and syphilis testing 20, could also be adapted in emergencies.

Collective outcomes should be defined from the onset of the emergencies, and a robust monitoring and evaluation system put in place to track progress towards SDGs. Sustaining peace resolutions, is critical for maintaining this progress. The renewed momentum in HIV workstreams spearheaded by the new post‐Global Plan programming framework, *Start Free, Stay Free, AIDS Free* 12, provides an opportunity for moving forward with this agenda. Creating a humanitarian‐development nexus is the centrepiece for the *new way of working* arising from the global humanitarian summit and a major theme in the Sendai framework for Disaster Risk Reduction 2015 to 2030 13. These are critical elements for achieving SDGs without leaving behind children and adolescents living with HIV in humanitarian crises.

## Competing interests

Authors declare no competing interests DC, CL and PI have not received grants or speakers fees from any commercial body in the preparation or submission of this manuscript

## Authors' contributions

DC conceptualized the article, established links between emergencies and HIV, and drafted the first draft. CL and PI reviewed the draft, elaborated links with SDGs and contributed to the writing of the manuscript
